# Monkeypox epidemiology, clinical presentation, and transmission: a systematic review

**DOI:** 10.1186/s12245-023-00491-3

**Published:** 2023-03-17

**Authors:** Ashima Sharma, Hari Prasad, Nidhi Kaeley, Aparna Bondalapati, Lokesh Edara, Y. Ajay Kumar

**Affiliations:** 1 Department of Emergency Medicine, Nizam’s Institute of Medical Sciences, Hyderabad, Telangana 500082 India; 2 Department of Emergency Medicine, All India Institute of Medical Sciences Rishikesh, Rishikesh, Uttarakhand 249203 India; 3 Department of Internal Medicine, Western Michigan University School of Medicine, Kalamazoo, MI USA

**Keywords:** Complications, Epidemiology, Monkeypox, Monkeypox virus, Transmission

## Abstract

**Background:**

The new zoonotic viral infection, monkeypox, is a global health issue. Our study aimed at studying the epidemiology, clinical presentation, complications, case fatality rate, and transmission among the present cases of monkeypox infection.

**Methods:**

Articles were searched in PubMed, Google Scholar, and Science Direct databases using the keywords “Monkeypox” [MeSH] or “Monkeypox virus” (MeSH). Narrative reviews, conference abstracts, commentaries, and articles in languages other than English were excluded.

**Results:**

From three databases, 1442 studies were identified. Seven hundred ten articles were excluded because they included data before 2022, leaving 732 items for screening. After filtering 320 data due to data duplication, 412 remained. Due to the inclusion of systematic reviews, meta-analyses, reviews, comments, and articles in languages other than English, 257 were excluded. Eligibility based on full-text review was applied to the remaining 155, excluding 129. So, the study covered a total of remaining 26 articles. We studied 2352 confirmed cases from published literature, accounting for approximately 4% of infected cases worldwide. Around 81.71% of patients have a bisexual or men having sex with men (MSM) preference. Approximately 30.18% of confirmed cases were HIV positive. Male sex was also identified as a risk factor in our review.

**Conclusion:**

Monkeypox human-to-human and human-to-animal transmission are rising. Thus, it is essential to do research on the prevention, clinicodemographic trends, and treatment of monkeypox. Understanding this will enable us to treat monkeypox patients with a targeted and focused approach.

## Introduction

Although the COVID-19 viral illness was still raging, the year 2022 saw the emergence of a novel viral virus — monkeypox. This zoonotic viral illness was active on the African subcontinent, with a few rare but epidemiologically intriguing cases being reported now and then in other areas of the world. It was first discovered in humans in the Democratic Republic of Congo in 1970 [1]. Since May, the spread has become more global, with more than 109 non-endemic countries reporting around 76,871 monkeypox cases as on October 28, 2022. The illness was viewed as an emerging threat, and the World Health Organization (WHO) labeled it a public health emergency of worldwide concern on June 23, 2022 [2]. The disease has resurfaced with serious dermatologic, respiratory, and neurological manifestations in immunocompromised populations.

## Material and methods

### Study design and method

The systematic review was done in accordance with the Cochrane collaboration and preferred reporting items on systematic reviews and meta-analysis guidelines (PRISMA).

### Data source and selection process

A thorough search was done on PubMed, Google Scholar, and Science Direct using the keywords “Monkeypox” [MeSH] or “Monkeypox virus” (MeSH). The systematic review included all the case reports, case series, and original articles from January 2022 to September 2022.

### Objective

The systematic review aimed to study the epidemiology, clinical presentation, complications, transmission, and case fatality rate among the present cases of monkeypox infection from the published literature.

### Inclusion and exclusion criteria

The review included articles containing data from January 2022 to September 2022 and was in full text. A thorough search was made to find full-text articles on monkeypox, fulfilling the above objectives. Narrative reviews, conference abstracts, commentaries, and articles in languages other than English were excluded.

### Study selection

The inclusion and exclusion criteria were followed throughout every stage of the screening and selection process. The full-text screening was done after the title and abstract screening. Two reviewers (AS, HP, and NK) undertook the screening.

### Data extraction

Following the exclusion of duplicate ineligible studies, the eligibility of the remaining publications’ full texts was assessed. The review’s goals and objectives were the basis for developing the data extraction form. The following information was extracted: study author(s), study year, study country, study population, number of cases with gender, age, travel history, sexual history, HIV status, smallpox vaccination, and clinical manifestations. AS, HP, NK, AK, LE, and AB did the data entry. Quality assessment tools were applied to evaluate the bias risk rather than to exclude low-quality literature.

### Data synthesis

Where sufficient data were reported, it was tabulated and evaluated.

### Case definitions

The ICMR (Indian Council of Medical Research) guidelines were adhered to define a suspected case, probable case, and confirmed case of monkeypox infection. A suspected case of monkeypox is defined as a person of any age with a history of travel to affected countries within the last 21 days presenting with an unexplained acute rash and one or more of the following signs and symptoms of swollen lymph nodes, fever, body ache, headache, and profound weakness. A probable case meets the case definition for a suspected case with clinically compatible illness and has an epidemiological link. A confirmed case of monkeypox is defined as a laboratory-confirmed one using the detection of unique viral DNA sequences either by polymerase chain reaction (PCR) and/or sequencing.

## Results

**Fig. 1:**
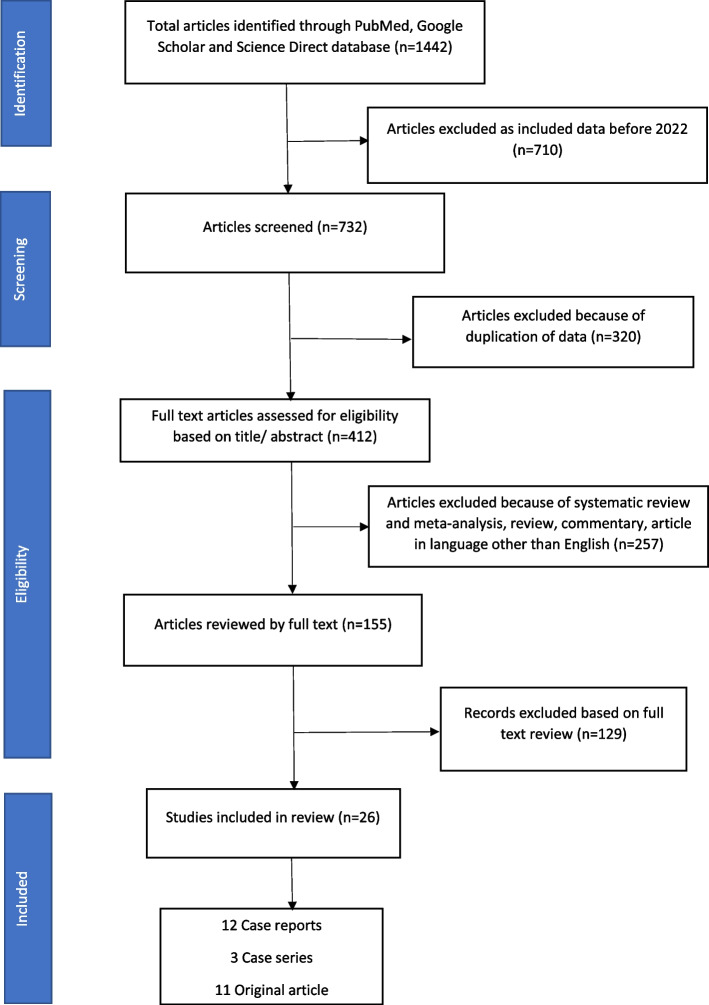
PRISMA flow chart

A total of 1442 studies were identified from three databases. Seven hundred ten were excluded as included data before 2022, giving a remaining 732 articles for screening. Four hundred twelve were left after excluding 320 because of data duplication. Two hundred fifty-seven were excluded because of included systematic review and meta-analysis, review, commentary, and article in languages other than English. Out of 155 remaining, eligibility based on full-text review was applied, which excluded 129. A total of 26 publications were included in the study comprising 12 case reports, three case series, and 11 original articles. A generous contribution to our review is from the news reports and websites of WHO, CDC, ICMR, and leading newsprint articles of various countries. Figure 1 shows the PRISMA flow chart. Table 1 shows the evolution of the monkeypox with important dates.

**Table 1:** Evolution of the disease with a few important dates

May 7, 2022	The first case in the UK, travel history from Nigeria
May 14, 2022	The next two cases, both in the UK, are unrelated to the first
May 19, 2022	Canada becomes the second country to report 17 suspects. The government also announced that the first suspect was identified on April 29, 2022, with a travel history to the USA Italy also shares the date of becoming the second country to report the first confirmed case
May 30, 2022	Nigeria reported the death of a confirmed case (first)
June 25, 2022	WHO emergency committee opined that monkeypox was not a public health emergency (PHE)
July 7, 2022	WHO corrects itself and considers monkeypox as PHE
July 8, 2022	The demand for monkeypox vaccine outstrips the supply, and the USA government website to facilitate vaccination crashed on July 13, 2022
July 15, 2022	CDC considered Tembexa, a drug used for smallpox for use despite multiple adverse effects The first case of monkeypox infection was confirmed in India
July 19, 2022	The infection was declared to be endemic in the MSM community
July 23, 2022	WHO recognized monkeypox as a Public Health Emergency of International Concern (PHEIC)
August 1, 2022	India reported the death of a confirmed case
August 15, 2022	A Spanish study suggested that the smallpox vaccine may not provide lifelong immunity from infection The first case of human-to-dog transmission was reported
August 16, 2022	Considering monkeypox as a misnomer, WHO opened the process of renaming the virus to the public
August 26, 2022	US doctors reported atypical or absolutely no symptoms in monkeypox infection
September 9, 2022	Neurological complications of monkeypox infection were reported
September 16, 2022	Caution against over-prescription of tecovirimat to prevent viral mutations

Though every day since May 2022 has been intensely monitored worldwide, especially in the non-endemic nations, a few dates have been crucial. The USA was the first nation, and Egypt is the most recent country to have reported its first confirmed case. Vaccine tourism was observed in August 2022, and interestingly, WHO made many turnabouts before recognizing the fatal potential of the infection.

Table 2 reports a death rate of 0.04% (23 deaths, with a significant 14 from Africa) with the present strain. However, it is very much possible that not all deaths are getting reported or captured. The USA, Spain, Brazil, France, Germany, the UK, Peru, Canada, Columbia, and the Netherlands account for 86.7% of cases worldwide. Also, a steady decrease in cases of almost 22% was reported in North America and Europe.

**Table 2:** Burden of cases in different continents (October 28, Reuters)

Continent	Total cases	The three countries with the highest number of confirmed cases	Deaths (number)
America	48,929	USA Brazil Columbia	USA 2/28379 Brazil 2/3110 Cuba and Ecuador 1 each
Asia	64	Singapore India Thailand	India 1/19
Australia	140	New Zealand	
Europe	24,950	Spain France Germany	Spain 2/7277 Czech Republic 1/70 Belgium 1/785
Middle East and Africa	332	Israel Lebanon UAE	Mozambique 1/1

We extracted information on only 4% of the confirmed worldwide cases of monkeypox infection regarding their gender and the median age of infection. The disease has been far more active in the male population (2332 men to 19 women and one transgender) with a median age of 35 years. A pediatric patient below 10 years of age and a woman at 71 years have also been reported. 81.71% of infected cases were from the MSM community. Table 3 shows details of patients with age, gender, travel history, sexual history, and HIV status.

**Table 3:** Demographics and exposure history from the available articles

Authors	Number of cases	Country	Gender	Median age	Travel history	Sexual history (gay/bisexual/MSM)	HIV status
Antinori et al. [3]	4	Italy	All males	30	Yes	All MSM	HIV + 2/4
Sukhdeo et al. [4]	1	Canada	Male	33	No	Yes-MSM	Negative
Jang et al. [5]	1	Korea	Male	34	Yes	Yes-bisexual	Unknown
Hammerschlag et al. [6]	1	Australia	Male	30	Yes	Yes-MSM	HIV +
Tutu van Furth et al. [7]	1	Netherlands	Male	10	Yes	Nil	Negative
Noe et al. [8]	2	Germany	Both males	26 and 32 years	No	Yes-MSM	HIV + 2/2
Vivancos et al. [9]	86	UK	Males, 79 Females, 7	38	Yes, 1/86	Yes, 66 gay/bisexual/MSM	Unknown
Perez Duque et al. [10]	27	Portugal	Males	33	Yes, 4	Yes, 18 MSM	HIV + 14/18
Thornhill et al. [11]	528	16 countries	Males, 527 Transgender, 1	38	Yes, 147	Yes, 509 MSM, 10 bisexual	HIV + 218/528
Iñigo Martínez et al. [12]	508	Spain	Males, 503 Female, 5	35	Yes, 38	Yes, 397 MSM	HIV + 225/508
Girometti et al. [13]	54	UK	All males	41	Yes, 25	Yes, 54 MSM	HIV + 13/54
Miura et al. [14]	18	Netherlands	All males	Age range 23–64	Nil	Yes-all MSM	Unknown
Català et al. [15]	185	Spain	All males	38.7	Yes-51	Yes, 184 MSM	HIV + 78/185
Oprea et al. [16]	1	Romania	Male	26	No	Yes-MSM	HIV +
Minhaj et al. [17]	17	USA	All males	40	Yes, 14	Yes, 16 MSM	HIV + 3/17
de Sousa et al. [18]	1	Africa	Male	24	Unknown	Yes-MSM	HIV +
Peiró-Mestres et al. [19]	12	Spain	All males	38.5	Yes, 4	Yes-all MSM	HIV + 4/12
Patel et al. [20]	197	UK	All males	38	Yes, 54	Yes, 196 gay/bisexual/MSM	HIV + 70/197
Tarín-Vicente et al. [21]	181	Spain	Males, 175 Females, 6	37	Nil	Yes, 166 gay/bisexual/MSM	HIV + 72/181
Mileto et al. [22]	1	Italy	Male	33	Yes	Yes-MSM	HIV +
Bížová et al. [23]	1	Czech Republic	Male	34	Yes	Yes-MSM	HIV +
Vallée et al. [24]	1	France	Male	Unknown	Unknown	Yes-MSM	HIV +
Raccagni et al. [25]	1	Italy	Male	42	Yes	Yes-MSM	HIV +
Bruno et al. [26]	1	Italy	Female	71	Nil	Yes, with multiple partners	HIV +
de Nicolas-Ruanes et al. [27]	1	Spain	Male	30	Nil	Yes-MSM	HIV +
Selb et al. [28]	521	Germany	Male	38	Nil	Yes, 259 MSM	Unknown

We had data on only 2352 confirmed cases from published literature, approximately 4% of infected cases worldwide. However, it can be commented that 81.71% of patients have a bisexual or MSM preference. None of the victims has identified exposure to animal carcasses. Around 30.18% of confirmed cases were HIV positive. The transmission seems, therefore, to be direct skin and mucosal contact during sexual activities in 81.71% of cases. History of smallpox vaccination was reported only in Perez Duque et al., Thornhill et al., Català et al., Peiró-Mestres et al., Tarín-Vicente et al., and Bruno et al. as 1, 49, 39, 4, 32, and 1 patients respectively among studies included in the review [10, 11, 15, 19, 21, 26]. Signs and symptoms in the majority of patients are shown in Table 4.

**Table 4:** Clinical features in the majority of the patients

Author	Clinical manifestations
Antinori et al. [3]	Asynchronous rash—genital area, inguinal lymphadenopathy; fever, asthenia, perianal lesions, inguinal lymphadenitis followed by anal lesions, lesions on feet, legs, back, suprapubic area and chest
Sukhdeo et al. [4]	Rectal pain, pustular rash involving the face, extremities, and torso; enlarged, painful, tender, cervical, and inguinal lymphadenopathy
Jang et al. [5]	Asymptomatic, perioral erosive lesions, tiny papules scattered across the back and lower abdomen, painless ulcer on the penile shaft, bilateral enlarged and tender inguinal lymph nodes, chills, sore throat, subjective fever followed by an extensive rash
Hammerschlag et al. [6]	Painless white pustules on his penis, fever, and malaise; the rash disseminated to his trunk, then sparingly to the face and limbs while the genital lesions crusted over
Tutu van Furth et al. [7]	Prodromal sore throat, gradually spreading lesions
Noe et al. [8]	Malaise, fever, joint pain, muscle and back pain, headache, dysphagia followed by white spots on tonsils
Perez Duque et al. [10]	Exanthem, inguinal lymphadenopathy, fever, and genital ulcers
Thornhill et al. [11]	Prodromal—fever, lethargy, myalgia, headache, lymphadenopathy; concomitant sexually transmitted illnesses followed by a rash, anogenital, and mucosal lesions
Inigo Martinez et al. [12]	Rash mainly anogenital, fever, asthenia, lymphadenopathy
Girometti et al. [13]	Fatigue or lethargy, fever, and skin lesions, with 51% of patients having anogenital lymphadenopathy
Català et al. [15]	Primary localized homogenous papules on skin or mucosa, generalized small pustules appeared later, mucosal ulcers and monkey whitlow less commonly
Oprea et al. [16]	Fever with chills, rectal pain, dysphagia, and vesiculopustular rash
Minhaj et al. [17]	Rash in the genital or perianal area, chills, fatigue, lymphadenopathy, fever
de Sousa et al. [18]	Disseminated papules throughout the trunk, face, and genital area and several clusters of umbilicated white papules in the shape of kissing lesions could be noticed on the perianal region
Peiró-Mestres et al. [19]	Fever, myalgia, generalized malaise, and skin lesions were present in more than one location of the body
Patel et al. [20]	Mucocutaneous lesions on genitals or perianal area, rectal pain, penile edema, sore throat, fever, myalgia, and lymphadenopathy
Tarín-Vicente et al. [21]	Skin lesions on anogenital, oral, and perianal areas, fever, headache, sore throat, and lymphadenopathy. In severe cases, proctitis and tonsillitis
Mileto et al. [22]	An ulcerated perianal lesion, two minor papular lesions on both elbows, asthenia, malaise, anorexia, lymphadenopathy, and a new lesion on the cheek that was accompanied by upper respiratory tract symptoms (sneezing and pharyngodynia)
Bížová et al. [23]	Fever, itchy rash on the forehead, and painless perianal erosions. Later, papulovesicular lesions on the forehead with lymphadenopathy appeared
Vallée et al. [24]	Fever with chills, fatigue, malaise, sore throat, anal pain, and lymphadenopathy
Raccagni et al. [25]	Diarrhea, tenesmus, two atypical nonvesicular erythematous cutaneous and perianal lesions
Bruno et al. [26]	Papules on buttocks, trunk, and umbilicus
de Nicolas-Ruanes et al. [27]	Initially started as proctitis, fever, headache, lymphadenopathy, generalized arthralgia, and myalgia. Later, on both arms and the dorsum of the hands, there was a maculopapular pinkish exanthem associated with many flat-topped umbilicated pustules with necrotic centers that later extended to the trunk, legs, face, and genitalia, sparing the palms and soles

The various presentations have suggested that rash and genital ulcers are seen in all patients. The presence of prodrome symptoms was subjective. Patients remained completely asymptomatic as well till the onset of ulcerative lesions. Hence, it is vital to have a high surveillance index in a particular community for early isolation and initiation of treatment.

The confirmation tests had predominantly been PCR of lesional fluid. In very few instances, multisite PCR has been used, showing variable results, with negative results from nasopharyngeal swabs but positive results in the pustular fluid. Punch biopsy of genital lesions and genome sequencing have also been tried modalities for case confirmation.

Tecovirimat was short in supply; therefore, in initial cases, it was not used for all patients. Empirical acyclovir was also utilized. Supportive treatment was done using antihistamines, anti-inflammatories, and topical zinc oxide.

Cases from India have presented with fever and an unexplained acute rash all over the body with or without headache, swollen lymph nodes, myalgias, backache, and profound weakness [29].

## Discussion

The systematic review was done to study the recent outbreak of monkeypox viral infection to understand the possible ways of preventing viral replication, transmission, and early recognition and treatment of the disease. The articles suggest that the monkeypox outbreak has spread globally to multiple endemic and non-endemic countries. In the recent attack, the first cases were identified in the UK in May 2022 [30]. There was no history suggesting travel to endemic areas or close contact with a person to have monkeypox. Adler et al. published a case series of seven patients infected with monkeypox over 4 years (2018–2021). They suggested that some sporadic cases were reported just before a major outbreak in the UK [31]. Another report from the CDC website suggests that 36–42% of HIV-positive patients get infected with the monkeypox virus. This information can be alarming for countries where HIV-infected individuals are significantly higher. Monkeypox is mainly transmitted from animal to human or human to human with close contact with direct skin lesions (microabrasions), bites, and fomite spread [32]. Transmission through large respiratory droplets is also suggested, especially with longer face-to-face contact time. There is also evidence of vertical transmission [33].

### Epidemiology

Since 1980, WHO was apprehensive about the possibility of an outbreak as the vaccinia vaccine for smallpox was discontinued [34]. Monkeypox is caused by the poxviridae family, which is a double-standard deoxyribonucleic acid virus. The poxviridae family further has two subfamilies, Chordopoxvirinae and Entomopoxvarinae. Monkeypox virus belongs to the chordopoxvirinae subfamily and the genus *Orthopoxvirus*. The other poxvirus species which cause human infections are varied (smallpox), cowpox, Alaska pox, Yaba monkey tumor virus, pseudocowpox virus, buffalo pox, and molluscum contagiosum viruses. The major hosts of poxviruses are rodents, rabbits, and non-human primates. It has also been commented that the virus must have evolved since past outbreaks, considering the new symptomatology, broader geographical spread, and diverse population involvement [35].

Non-immunization with the smallpox vaccination, residing in forested regions, male sex, age less than 15 years, and high-risk sexual preferences are all associated with an increased risk of infection [36]. Male sex was also identified as a risk factor in our review.

### Clinical features

Clinically, in the present outbreak, patients commonly have genital and/or oral ulcers without systemic illness [6, 7]. The systemic manifestations are fever, headache, sore throat, back pain, and fatigue. These can also occur after the rash appears; however, more commonly, they are seen before the rash. The incubation period is 6 to 13 days, extending from 5 to 21 days [14]. The rash characteristically starts as 2–5-mm macules and progresses to a papule, vesicle formation, and pseudo pustular stages in a week to 2 weeks. These pseudo pustules are well-circumscribed, deep-seated, and umbilicated. The main sites involved are anogenital and perioral areas with few lesions on the trunk and limbs in the present outbreak. The genital lesions in men can present with paraphimosis and necrotic crusts. The rectal lesions cause proctitis with pain on defecation as the presenting symptom. Oral mucosa, tongue, pharyngeal wall, and tonsils can have ulcers with central depression. The rash is similar in appearance to smallpox, secondary syphilis, herpes simplex, and chickenpox infections [11].

### Complications

The reported complications of monkeypox infection are bronchopneumonia, sepsis, encephalitis, myocarditis, keratitis with visual loss, rectal proctitis, and rectal wall perforations. The most common complication is coalescing of large ulcers with superimposed cellulitis. Pediatric patients and patients with immunocompromised status can have severe forms of the disease [12]. Two different genetic clades of the virus have been identified: the Central African (Congo Basin) and West African clades. The Congo Basin clade is more virulent and responsible for more severe diseases. The deletion and fragmentation of the open reading frame of the West African clade are responsible for the lower virulence of the clade. Another reason for the increased virulence of the Congo Basin clade is that monkeypox inhibitor of complement enzymes is absent in the West African strain. The human-to-human transmission was found to be sustainable, with a reproductive ratio (Ro) of 1.29 (1.10–2.40) [37–39].

### Pathophysiology

Poxviruses are large viruses and have double-stranded DNA. They replicate in the cytoplasm of both vertebrate and invertebrate cells. The large ortho poxviruses are able to stimulate early immune response with CD8 + cells. The infection is self-limiting as the subsequent production of gamma interferons IFN-γ, IL-1rs, IL-6, IL-8, and MCP-1 stops viral replication. The laboratory diagnosis on a suspected case is done using PCR testing for orthopox virus DNA performed on the lesion fluid, ELISA for anti orthopox virus IgM and IgG detected during 5–8 days after onset of rash, respectively, and electron microscopy to identify brick shaped pox virus virions [40].

### Investigations

Laboratory investigations may reveal abnormal aminotransferases, leukocytosis, thrombocytopenia, and hypoalbuminemia [36]. The management of monkeypox is mainly supportive. The patients may require hospitalization for isolation, pain management, and treatment of secondary complications. Mild cases should be assessed for the analgesic requirement for proctitis. Stool softeners, topical lidocaine, and sitz baths should be the first line of treatment. They should also get instructions to report to the hospital if symptoms of dehydration (nausea, vomiting, dysphagia, severe tonsillitis), intravenous pain medications are required, and/or the patient develops any severe complications [41].

### Treatment

The most common drug used for monkeypox infection is tecovirimat, which is an inhibitor of the orthopoxvirus VP37 envelope wrapping protein. Patients with severe disease or at risk of progressing to a severe illness were young (less than 8 years), atopic dermatitis, exfoliative skin lesions, pregnancy, and breastfeeding females. Patients with atypical infection sites and eye or eyelid involvement should also receive tecovirimat. Although there is no data on the efficacy of tecovirimat in treating people with mpox, investigations on a range of animal species have demonstrated that tecovirimat is useful in treating illnesses caused by orthopoxviruses. The role of the vaccinia vaccine is not proven therapeutic. The vaccination window for these contacts was set to be within 4 days of exposure, with a maximum of 14 days [42].

The patient should be kept in a single room with door isolation to prevent spread inside the hospital. It may not be an airborne infection isolation room. The patient should be instructed to wear a mask if other people are in the patient’s room. The healthcare staff should exercise contact precautions and wear an N95 mask during rounds. An accidental exposure should mandate an observation/isolation for 21 days [43].

### Limitations

This systematic review has some limitations. First, our ability to present a complete picture of the number of confirmed cases was sometimes limited, as data quantity and quality varied across regions. Most studies are from Europe, South, and North America. Only a few studies are from Asia, Africa, and Australia. So, the data is not uniform. Second, sexual history and HIV status are reported in most of the studies. There was a paucity of data on HIV status in some studies. Some have either not performed the HIV testing or the patient may be in a window period at the testing time. So, the exact burden of monkeypox patients with HIV has to be studied. Third, the history of other sexually transmitted diseases (STDs) is not included in the review. As monkeypox’s major transmission route is sexual, STD coinfection is expected.

## Conclusion

Before the population has had a chance to heal from the effects of a previous virus, a new one strikes. This is the case with monkeypox and COVID-19 infection. The preliminary sequencing data (15 isolates) reveal that the monkeypox virus is experiencing faster human adaptation due to higher than predicted changes in the DNA genome. This emphasizes the critical importance of researching preventative and therapeutic solutions for the monkeypox outbreak. The vast majority of patients are MSM, with a greater prevalence of HIV infection. Male gender has been recognized as a risk factor as well. The review improves understanding of monkeypox’s clinicodemographic, preventative, and therapeutic aspects. Knowing this will allow us to treat patients in a more targeted and organized manner.

## Data Availability

The datasets generated during and/or analyzed during the current study are available from the corresponding author on reasonable request.
